# Complex Analysis of Retroposed Genes’ Contribution to Human Genome, Proteome and Transcriptome

**DOI:** 10.3390/genes11050542

**Published:** 2020-05-12

**Authors:** Magdalena Regina Kubiak, Michał Wojciech Szcześniak, Izabela Makałowska

**Affiliations:** Institute of Human Biology and Evolution, Faculty of Biology, Adam Mickiewicz University, 61-614 Poznań, Poland; magdalena.kubiak@amu.edu.pl (M.R.K.); miszcz@amu.edu.pl (M.W.S.)

**Keywords:** retroposition, pseudogenes, lncRNA, miRNA sponge, antisense transcript, recombination

## Abstract

Gene duplication is a major driver of organismal evolution. One of the main mechanisms of gene duplications is retroposition, a process in which mRNA is first transcribed into DNA and then reintegrated into the genome. Most gene retrocopies are depleted of the regulatory regions. Nevertheless, examples of functional retrogenes are rapidly increasing. These functions come from the gain of new spatio-temporal expression patterns, imposed by the content of the genomic sequence surrounding inserted cDNA and/or by selectively advantageous mutations, which may lead to the switch from protein coding to regulatory RNA. As recent studies have shown, these genes may lead to new protein domain formation through fusion with other genes, new regulatory RNAs or other regulatory elements. We utilized existing data from high-throughput technologies to create a complex description of retrogenes functionality. Our analysis led to the identification of human retroposed genes that substantially contributed to transcriptome and proteome. These retrocopies demonstrated the potential to encode proteins or short peptides, act as *cis-* and *trans*- Natural Antisense Transcripts (NATs), regulate their progenitors’ expression by competing for the same microRNAs, and provide a sequence to lncRNA and novel exons to existing protein-coding genes. Our study also revealed that retrocopies, similarly to retrotransposons, may act as recombination hot spots. To our best knowledge this is the first complex analysis of these functions of retrocopies.

## 1. Introduction

Over the last decade the way we look at the human and other genomes has changed in a striking way. For instance, the estimated fraction of the human genome derived from retroposition has increased to over 70% [1]. Furthermore, the discovery that these sequences, considered as a “junk DNA”, may play a crucial role in shaping genome-specific features was one of the most surprising breakthroughs of human and others genomes analyses. It has also been established that the majority of human RNA transcripts do not encode proteins and that non-coding RNAs regulate cell functions. These discoveries strongly support an RNA-centric view of evolution in which phenotypic diversity arises through extensive RNA processing and an RNA-directed rewriting of DNA. One of these processes, which plays a fundamental role in evolution, is the birth of new genes via retroposition. In this type of gene duplication, multi-exon genes give birth to single-exon copies that, in most cases, lack regulatory elements and are commonly believed to be pseudogenes [2]. 

Although the majority of copies of protein-coding genes generated by reverse transcription are in a state of “relaxed” selection and remain “dormant”, as they lack regulatory regions, many of them are known to recruit new regulatory regions [3] and produce new genes [4,5,6]. Therefore, retro(pseudo)genes, copies of parental genes, which for a long time were considered not to be important, are nowadays called “seeds of the evolution” [7]. It has been shown, by us and other groups, that they made a significant contribution to molecular evolution and played an important role in the diversification of transcriptomes and proteomes. Retrocopies of protein-coding genes may be responsible for a wealth of species-specific features [4,8,9]. A very elegant example of the functional phenotypic effect of gene retroposition is retrogene *fgf4*, which is responsible for chondrodysplasia in dogs. All breeds with short legs are carriers of the *fgf4* retrogene [10]. Another interesting example is the functional mouse retrogene *Rps23r1*, which reduces Alzheimer’s beta-amyloid levels and tau phosphorylation [11]. This particular retrogene is rodent specific and does not exist in the human genome. A study of the tumor-suppressor gene *TP53* suggests that gene duplicates, which arose via retrotransposition, play a role in the reduction of cancer risk in elephants [12]. 

**Figure 1:**
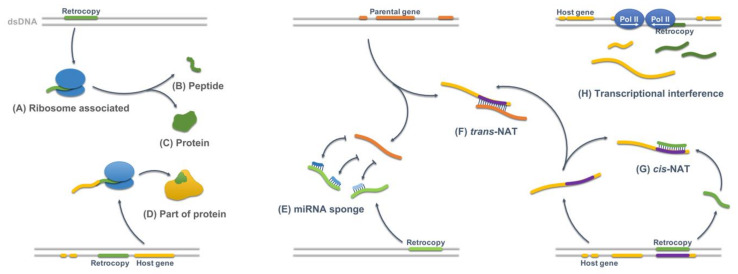
Different manners of retroposed genes contribution to proteomes and transcriptomes. Retrocopies may associate with ribosomes (**A**) and encode short peptides (**B**) or proteins (**C**). It has been also demonstrated that retrocopies contribute novel exons to existing genes (**D**). They may also regulate their parental and other genes expression as miRNA sponges (**E**), *trans*-NATs (**F**), and *cis*-NATs (**G**). Nested retrocopies might also regulate splicing of their hosts via transcriptional interference (**H**).

The evolutionary path of functional retrogenes is not uniform. In the course of evolution they may undergo subfuctionalisation and consequently share the function with their parent [13] or, as our studies have shown, replace their parental gene [14]. Nevertheless, as duplicates of their progenitors, the majority of retrocopies evolve relatively fast because duplication events allow a relaxed purifying selection, and these genes may acquire novel functions (neofunctionalisation) [13,15]. These functions come from the gain of new spatio-temporal expression patterns, imposed by the content of the genomic sequence surrounding inserted cDNA [16], and/or by selectively positive mutations which may lead to the switch from protein coding to regulatory RNA [17,18]. As studies in *Drosophila melanogaster* have shown, these genes may very quickly become essential [19]. They can also lead to new protein domains formation through fusion with other genes [20,21], new regulatory RNAs [17,22], or other regulatory elements [23] (Figure 1).

Retrocopies of protein-coding genes are also known to be involved in many diseases. A good example is the *RHOB* gene, a tumor suppressor of the Rho GTPases family, which arose by retroposition in the early stage of vertebrate evolution [24]. Mutation in another retrogene, *TACSTD2* — tumor associated calcium signal transducer 2, causes a gelatinous drop-like corneal dystrophy which leads to blindness [25]. Our study has shown that out of 25 retrogenes, which have replaced their progenitor, seven are associated with human diseases, including cancer, diabetes, attention-deficit/hyperactivity disorder, Huntington’s disease, and others [14]. However, none of these retrogenes have been previously recognized as a retrocopy of decayed or deleted gene.

Although analysis is challenging due to the high similarity to parental genes and a low expression level, the attention given to these duplicates commonly considered as pseudogenes is recently growing [26,27,28,29]. They are especially interesting for those studying cancer since a number of pseudogenes were already proven to be promising biomarkers [30,31,32]. 

The development of high-throughput methods has enabled a wide characterization of genomes, transcriptomes, and even epigenomes of various organisms, organs and tissues. These experiments provide information about gene expression, methylation patterns, association with ribosomes and much more. We utilized these vast amounts of data to test which of retrogene-derived RNA transcripts may possess an active biological role and to create a detailed description of retrogenes functionality. All possible functions demonstrated on Figure 1 were investigated in the course of this study. Although some of the performed analyses were conducted also by other authors, this is the first such complex analysis taking into consideration a variety of possible functions focused on human retrocopies of protein-coding genes. This analysis has led to the identification of over two thousands retrocopies which have contributed to human transcriptome and proteome. Moreover, our studies revealed, for the first time, that retrocopies have unusually high expression in the spleen and that, similarly to retrotransposons, may act as recombination hot spots. 

Retroposed genes until recently were commonly named processed pseudogenes or retropseudogenes under the assumption that they are not functional. Here we refer to all duplicates that originated by means of retroposition as retrocopies since they represent both pseudogenes and novel genes resulting from RNA-mediated duplication [13]; to the functional retrocopies we refer as retrogenes.

## 2. Materials and Methods 

### 2.1. Re-Annotation of Retrocopies

The human retrocopy repertoire from the RetrogeneDB [33] was used as a source of basic retrocopy annotations. The database contains 4611 human retrocopies, of which 106 have a known protein-coding status, 4384 are annotated as known pseudogenes and 121 as novel, meaning not previously annotated in other databases. However, the RetrogeneDB was built on a former human reference genome version (GRCh37). As a newer release of the reference human genome is available (GRCh38), we lifted over coordinates of retrocopies between assemblies. To this goal we used an online Assembly Converter by Ensembl [34]. A final dataset of 4555 successfully mapped retrocopies was obtained.

### 2.2. Retrocopies Expression Analysis

Paired-end reads with RNA sequencing results were downloaded from ENCODE, totaling 818 experiments. The RNA-Seq reads were subjected to quality filtering, quality trimming and adapter clipping with BBDuk2 from BBTools package (Joint Genome Institute; https://jgi.doe.gov), using the following settings: qtrim = w, trimq = 20, maq = 10, rref = adapters.fa (a built-in set of Illumina adapters), k = 23, mink = 11, hdist = 1, tbo, tpe, minlength = 50, removeifeitherbad = t. The parameters are explained on the tools website [35]. To remove rRNA-derived reads, mapping against a set of human ribosomal RNAs from Ensembl and RefSeq was performed with Bowtie 2 [36] and only unmapped reads were kept. Expression of each gene and transcript was estimated with Salmon v0.9.1 [37] using default parameters, except for—seqBias and—gcBias, to correct for potential sequence-specific biases in the input data. Only transcripts expressed at a minimum of 1 TPM (Transcripts Per Million) in at least 1% of the experiments were kept.

### 2.3. Expression correlation

From the whole transcriptome, we extracted expression values of retrocopies, parental genes, and potential trans-NATs. Spearman correlation coefficients for transcripts and retrocopies as well as parental genes and potential trans-NATs were calculated using the Python library SciPy [38]. We assumed that in case of trans-NATs and miRNA sponges’ predictions, the potential functions are performed on a transcript level. Therefore, we calculated the expression correlation only in experiments in which transcripts were co-expressed. In case of *cis*-NATs and transcriptional interference predictions, function can be performed not only at a transcript level, but also during the transcription process itself. In this scenario, the transcription of one gene may have a suppressive influence on a transcriptional process of a second gene. Thus, we did not remove any information from the input file, even when the level of expression was equal to 0 TPM. We required that the *p*-value was below 0.001 and the correlation coefficient (rho) was higher than 0.25 or lower than −0.25 to consider transcripts expression as correlated. 

### 2.4. Conserved Domain Analysis

Conserved protein domains were analyzed using the Batch CD-search tool [39]. To compare the domain composition of retrocopies and parental genes, resulting datasets were collated in one file and manually curated.

### 2.5. Gene Ontology Analysis

The PANTHER Classification System [40] was used to categorize proteins encoded by retrocopies according to Gene Ontology terms such us molecular function, biological process, and cellular compartment. The PANTHER home page provides access to the gene list analysis tool which supports Ensembl identifiers. Identifiers of retrocopies annotated in Ensembl as protein-coding genes were utilized as an input file. The functional classification and statistical overrepresentation test with default settings were selected to analyze the data. Fisher’s exact test with the Benjamini-Hochberg False Discovery Rate (FDR) was computed and only categories with FDR lower than 0.05 were taken into consideration as statistically significant.

### 2.6. Mass Spectrometry-Based Proteomics Data Analysis

A set of peptides was obtained from PRIDE [41] We have omitted peptides shorter than ten amino acids (aas). The retrocopy coordinates were extended by 500 bases at both ends. FASTA sequences were fetched with bedtools getfasta. Afterwards, peptides from the PRIDE database were mapped against retrocopies with TBALSTN using an e-value of 1000 as a threshold. To reduce a false positives rate, the mapped peptides were also compared against cDNAs and ncRNAs from Ensembl. Importantly, the list of peptides has been limited to those that match retrocopies with 100% sequence identity along the entire length of the peptide (no gaps and mismatches were allowed). Analysis was done for six open reading frames (ORFs). It was required that the ORFs overlap with at least 60 bases (20 aa) of the original retrocopy coordinates from RetrogeneDB. Only peptides that were mapped to valid ORFs were kept, making up the final set of peptides.

### 2.7. Identification of Ribosome-Associated Retrocopies

To identify ribosome associated retrocopies we utilized uniquely mapped reads from 26 Ribo-Seq and RNA-Seq experiments stored in GWIPs-viz Browser [42]. We used Elongating Ribosomes (Footprints) and mRNA-seq Reads global aggregate files in bigwig format, which were converted into bedGraph format with bigWigToBedGraph [43] and then to a BED file using a custom awk command. Genomic coordinates of CDSs and 3′UTRs of representative transcripts of protein-coding genes were utilized as positive and negative controls, respectively. Then, a bedtools intersect was applied to assign reads to retrocopies, CDSs and 3′UTRs. Finally, we have calculated a mean coverage of sequences by the RNA-Seq and Ribo-Seq reads and, following methodology by Zeng et al. [44], we further estimated a ribosome density. We used the following Equation (1):

(1) $$ribosome\;density=\frac{Ribo\;ratio}{RNA\;ratio}$$

where Ribo ratio is a mean coverage of sequence by Ribo-Seq reads and RNA ratio represents a mean coverage of a sequence by RNA-Seq reads. Only sequences with a mean coverage by RNA-Seq reads of at least 10 were taken into further consideration. In each group we have removed outliers based on a Z-score of −1.64 and 1.64, thus retaining 90% of the data. A Z-score equal to 1.64 calculated for 3′UTR dataset was used as a cut-off value for selecting ribosome associated retrocopies. All data processing and calculations were performed using in-house Python scripts. 

### 2.8. Identification of Retrocopies Overlapping With Other Genes, Trans-NATs, and Contributing Sequences to the Host Gene

Identification of retrocopies overlapping with other genes, trans-NATs, and contributing sequences to the host gene was performed based on genomic coordinates using BEDTools [45] and local Python scripts.

### 2.9. Identification of miRNA Sponges

In the first step of the miRNA sponges analysis Miranda software [46] with default parameters for miRNAs target prediction was used. The set of mature 2654 miRNAs from miRbase [47] and transcripts expressed at a minimum of 1 TPM in at least 1% of the experiments were included in the analysis. Nucleotide sequences of chosen transcripts were obtained from the Ensembl database [48]. The miRNAs target prediction was performed separately for the transcriptome and the set of retrocopies. Pairs of retrocopies and transcripts with common miRNA target sites were extracted. A hypergeometric test [49] was performed with help from the python library SciPy [38] to evaluate the probability of a retrocopy playing a role of miRNA sponge. Next, a Benjamini-Hochberg correction with α = 0.05 was performed using the python library StatsModels [50]. The data was complemented by an expression correlation estimation between retrocopies and other genes.

### 2.10. Identification of Fusion Transcript

Combined fusion breakpoint information from three representative fusion gene resources including ChiTaRS 3.1 [51], TumorFusions [52] and TCGA fusions [53] were acquired from the FusionGDB database [54]. Python scripts were used to filter the dataset in order to identify fusions between parental and host genes as well as between host genes of retrocopies originated from the same parental genes. Afterwards, the position of breakpoints was compared with the retrocopy location to determine the nature of retrocopy contribution in fusion transcript formation.

### 2.11. Data Processing, Filtering and Visualization

If not stated otherwise, data and resulting files analyses were performed by awk commands and/or scripts using the Python Data Analysis Library (pandas) [55]. Data was visualized by in-house scripts based on a statistical data visualization library called Seaborn [56].

## 3. Results and Discussion

### 3.1. Number of Retrocopies in the Human Genome and Their Localization

Analyzed retrocopies were downloaded from a RetrogeneDB database, a repository of retrotransposed genes identified in 62 animal and 37 plant species [33]. As for the human genome, RetrogeneDB includes 4611 retrocopies from which 4384 are annotated as known pseudogene, 106 have a status of known protein-coding genes and 121 as novel (i.e., these retrocopies are not annotated in any other database including Ensembl). The database was built on a previous human reference genome version (GRCh37) and annotated following Ensembl Release 73 [57]. As the GRCh38 is an improved representation of the human genome, in which many gaps were closed and sequencing errors corrected, we performed a multistep reannotation of human retrocopies. Consequently, we obtain set of 4554 retrocopies including 4351 known pseudogenes, 111 known protein-coding, 77 novel, and fifteen retrocopies with other Ensembl Release 85 statuses [48] (Table S1). What is interesting is that out of 107 novel retrocopies, which were successfully converted into GRCh38 from GRCh37, 30 are now present in Ensembl Release 85 and are annotated as processed pseudogenes, which supports our method for retrogenes identification applied in RetrogeneDB.

### 3.2. Patterns of Retrocopies Expression

**Figure 2:**
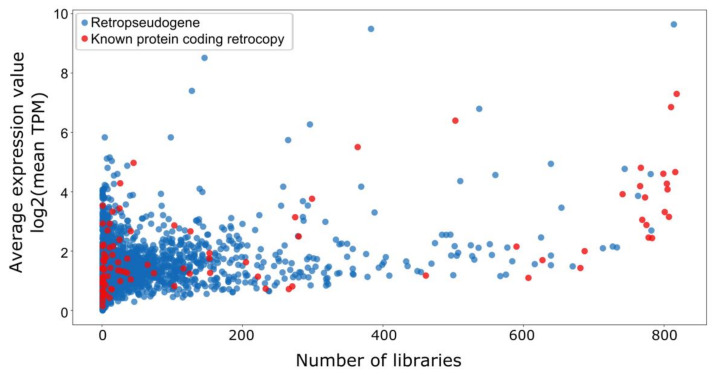
Expression of retrocopies. Retrocopies annotated as protein-coding genes are marked in red and other retrocopies are marked in blue. For each retrocopy the number of libraries in which it is expressed and the average expression value are marked.

Our previous analysis of RNA-Seq data, based on sixteen human tissues from the Illumina Bodymap 2.0 project, revealed that 579 retrocopies are transcriptionally active [33]. In this study we utilized ENCODE data [58] encompassing 818 experiments and representing a wide diversity of tissues, cell lines and experimental settings. As many as 1556 expressed retrocopies, annotated as pseudogenes, were identified. To consider a retrocopy as expressed, a minimum of 1 TPM (transcripts per million) uniquely mapped to the retrocopy was required. This may seem to be low. However, pseudogenes are known to have a relatively low expression. To overcome a problem with possible false positives due to the requirement of only 1 TPM expression, it was also required that this minimal level of expression should be detected in at least 1% of analyzed experiments, i.e., nine or more samples (Table S2). 384 retrocopies demonstrated transcriptional activity in 100 samples or more and 42 in over 50% of samples (Figure 2). A high number of retrocopies that were identified as transcriptionally active are predominantly expressed at a low level and in a limited number of samples (Figure 2). Nevertheless, out of 834 retrocopies expressed only in 1–5% of libraries, as many as 130 demonstrated an average expression over 10 TPM. 

**Figure 3:**
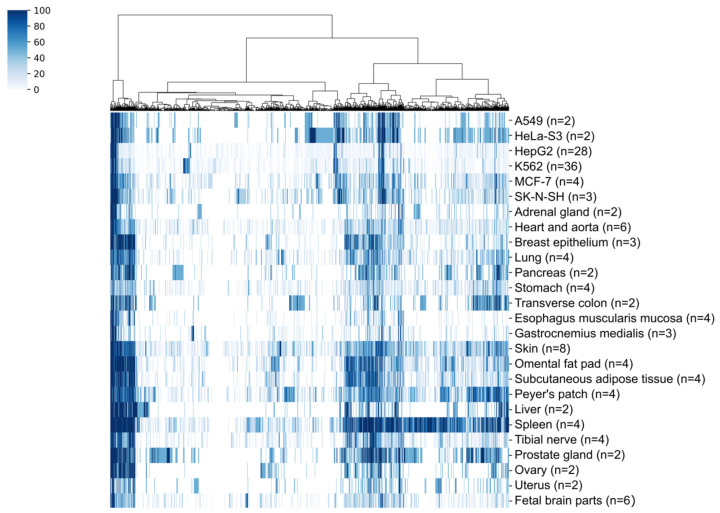
Percentage of samples from normal tissues and cancer lines in which retrocopies are expressed. A very dark blue color indicates that a given retrocopy is expressed in all samples of a particular type.

To analyze the pattern of retrocopies expression, samples from healthy tissues were selected and the percentage of samples in which the given retrogene is present was calculated with the requirement that at least two samples represent a given tissue (Figure 3). Considering that the number of retrocopies was identified as important in cancer and that the interest in pseudogenes as potential biomarkers is growing, similar analysis was performed for samples from cancer cell lines. The condition for including a particular cancer sample was that the sample is a control and was not subjected to experiments. The highest number of expressed retrocopies was detected in the spleen. As many as 420 retrocopies are expressed in all spleen samples. The lowest number of expressed retrocopies is in gastrocnemius muscle and esophagus muscularis, which is also in concordance with the expression level. Two cancer cell lines, chronic myelogenous leukemia (K562) and hepatocyte carcinoma (HepG2), appear to have the highest number of expressed retrocopies. However, most of them express in these cell lines sporadically and only a few are observed in more than 50% of samples. Fourteen retrocopies are ubiquitously expressed and they are present in all analyzed samples and 40 retrocopies demonstrated tissue specific expression (Table 1). The highest number of tissue-specific expressed retrocopies, fourteen, were identified in the spleen.

Previous studies demonstrated that retrocopies are especially highly expressed in the testis [5]. In our set we did not have any sample from the testis, however expression in the spleen seems to be also very high and this has not been previously reported. Pseudogenes were also formerly analyzed in various cancers. An example could be the identification of pseudogenes related to lung carcinoma [59], breast carcinoma [60] or other cancer types [28]. Here, a comparison of normal tissue and cancer cell lines revealed that three retrogenes are expressed in all analyzed cancer cell lines but not in normal tissues (Table 1). Identification of such retrocopies may be very valuable for tumor biology studies.

### 3.3. Retrocopies Potential for Protein and Peptides Coding

Retrocopies of protein-coding genes could contribute to the human proteome in several ways. They could quickly gain regulatory elements, before mutations accumulate, and have the same or similar functions as their progenitors. They could also act as lncRNAs that encode for short peptides. lncRNA’s capability to encode peptides has already been previously demonstrated [61]. It is also known that these peptides may play various regulatory roles [62]. Moreover, we may not exclude that these transcriptionally active retrocopies code for completely novel proteins. Finally, as it has already been indicated before, retrocopies may provide sequences for novel exons [21].

#### 3.3.1. Known Protein-Coding Retrogenes

Based on RetrogeneDB data and ENSEMBL annotations (Release 85) 111 known protein-coding genes were identified as originated via retrotransposition (Table S3). To investigate functions of these genes and to compare them with functions of their progenitors Gene Ontology analysis using PANTHER [40] was performed. The distribution of molecular functions and biological processes did not differ significantly between parental genes and their retrocopies (Figure S1). However, in the cellular component category the term “cell junction” (GO:0030054) was found only in the set of retrogenes. This term was assigned to *ARF6* (retro_hsap_36), a retrocopy of *ARF3* gene. Both genes encode ribosylation factors, factor 6 and 3, respectively, and play important roles in cytokinesis [63]. Both proteins are also described as activating the cholera toxin [64]. Nevertheless, some functions are attributed specifically to the *ARF6* retrogene. It has been recently demonstrated that it plays an important role in cell-cell interactions (adhesiveness) [65]. Interestingly, a number of studies indicate a strong association of ARF6 protein with human immunodeficiency virus type 1 (HIV-1) [66,67]. These two specific functions indicate a noteworthy subfunctionalisation process. Retrogene specific (i.e., not attributed to the parental gene) GO terms were also detected in the case of several other genes; however, these were lower level terms and could represent some annotation bias.

To further investigate signals for sub— or neofunctionalisation conserved domains were identified and subsequently compared in each retrocopy-parental gene pair. In 68 pairs there were no significant differences within the identified conserved domains and/or superfamily clusters. In the remaining cases some differences in domain composition were found and these pairs were manually inspected. In most instances, domains in retrocopies were identified only at the level of superfamily. Nevertheless, the superfamily was consistent with the respective parental gene. This may indicate changes in the retrocopy sequence leading to a loss of some signatures in functional domains. Detected were also pairs in which both genes have domains from the same superfamily cluster. However, specific domains differed. An interesting example is retro_hsap_28, also known as *RHOG*, and its parental gene *RAC1*. Domains of RHOG and RAC1 proteins are in the same superfamily called P-loop containing nucleoside triphosphate hydrolases (cl38936). Specific domains with different identifiers were found for RHOG and RAC1, cd01875 and cd01871, respectively. It is known that both of them have GTP/Mg^2+^ binding sites. However, it was shown that despite high sequence similarity between RHOG and RAC1, they diverge in specific residues that determine binding of effectors. For instance, it was experimentally confirmed that RAC1 binds PAK1 kinase, while RHOG does not. Instead, RHOG interacts with engulfment and cell motility protein (ELMO) and forms complex with dedicator of cytokinesis protein (DOCK1). The RHOG-ELMO-DOCK1 pathway is required for activation of RAC1 [68]. It has been also shown recently that it is possible that RHOG is an important player in Rac1-mediatet phagocytosis in human trabecular meshwork cells [69]. Another notable example is retro_hsap_105, known as *SLC5A3*, and the parental gene *SLC5A1*. Both genes contain solute binding domains. However, protein encoded by the retrocopy is a sodium-dependent mio-inositol transporter [70], while the protein encoded by the parental gene is involved in the active transport of glucose and galactose into cells [71]. Performed analysis also demonstrated that proteins encoded by retro_hsap_67, known as *PTTG2*, and by the parental gene *PTTG1* contain a securin domain (pfam04856). However, it has been shown that PTTG1, but not PTTG2, interacts with separase, a protein involved in separation of sister chromatid at the onset of anaphase [72]. The proteins are also distributed differently in the cell, while PTTG2 is found in nucleus and cytosol, PTTG1 is more concentrated in the nucleus [73].

Whether these and other instances of slight changes in the function and/or the localization should be considered as subfunctionalization or as neofunctionalization is a matter of debate. Nevertheless, even not very large changes are classified by some as neofunctionalization [74]. In any case, these examples demonstrate that retrocopies could be fully functional and perform functions that, at least at some level, differ from those assigned to their progenitors.

#### 3.3.2. Retrocopies Capability for Peptides Coding

To further investigate the coding potential of retrocopies the data deposited in PRIDE database [41], a repository of mass spectrometry-based proteomics data, was analyzed. A multistep BLAST-based approach allowed us to identify 2378 peptides that uniquely matched 740 retrogenes with 100% identity (Table S4). Interestingly, eighteen of these peptides matched respective retrogene in reverse orientation. Although in the majority only one or two peptides matched a given retrocopy, in the case of 75 retrocopies there were at least five peptides matching in the same frame. This may indicate a potential for encoding longer products. The highest number of matching peptides, nineteen, was found for the retro_hsap_3164. This retrocopy is expressed in thirteen libraries and demonstrates 87% similarity, with no stop codons or frameshifts, when compared with a protein encoded by its parental gene. Peptides that uniquely matched the retro_hsap_3164 covered 26% of translated ORF. The length of the retrocopy ORF is identical to the ORF of parental gene. In the case of seven retrocopies more than ten uniquely match peptides were identified. One of these retrocopies, retro_hsap_2401 had a significant expression signal in as many as 388 libraries. Matching peptides, although specific for this retrocopy, are in frame that agrees with the translation of the parental gene. The level of similarity between protein encoded by the parental gene and the putative protein encoded by retrocopy is only 80% suggesting at least some differences in function. This retrocopy is annotated as lncRNA *MSL3P1* and was identified as a renal cell carcinoma biomarker [75]. It was also associated with hair graying [76] and migraines [77]. Considering all evidence, it cannot be ruled out that this retrocopy has a double function: it encodes a protein similar to the one encoded by the parental gene and also acts as a regulatory lncRNA.

As already mentioned, eighteen peptides matched retrogenes in the frame opposite to the translation of the parental gene. Some examples are retro_hsap_58, a known protein-coding gene *UBQLN2* associated with lateral sclerosis [78], retro_hsap_903, which contributed sequence to the alternative exon at the 3′ end of lncRNA *RAB30-DT-210* located on the opposite strand of DNA, and retro_hsap_2190 that provided an alternative exon to one of the splicing forms of lncRNA *AC104131.1-201,* also located on the opposite strand of DNA. Peptides matching these two last retrogenes are translated in the orientation corresponding to the lncRNAs. Therefore, it is conceivable that these lncRNAs are actually coding.

Interestingly, in dozens of cases specific for retrocopies peptides were in the same reading frame as in the case of the parental gene despite the fact that ORFs in retrocopies are disturbed by a frameshift and/or stop codons. The best example of this is probably retro_hsap_1530, which is expressed in 26 libraries and matches eleven peptides that demonstrate 100% identity exclusively to this retrocopy. Interestingly, translation of the retrocopy sequence revealed a stop codon between two regions to which peptides matched. Still, all peptides are translated in the same reading frame as in the parental gene. This could be explained by the presence of introns, but a manual analysis of sequencing reads confirmed a single exon structure.

Analysis of mass spectrometry data led to the identification of as many as 740 retrogenes with uniquely matching peptides. This is a significantly higher number than in other studies investigating coding potential of pseudogenes. Utilizing data from a high resolution Fourier transform mass spectrometry Kim et al. identified 140 pseudogenes with a unique peptide sequence, i.e., different at least in one position from a sequence encoded by a parental gene [79]. These peptides which uniquely match retrogenes may indicate pseudogene transcription and its function at the protein level, which is still a rarely considered possibility. Of course, there is also a chance that at least some of these signals may have resulted from bogus translations with no function. Considering this, Xu and Zhang [80] used a different approach to pinpoint pseudogenes with a function at the protein level: comparison of the nonsynonymous/synonymous substitution rate ratio (ω) between putatively translated pseudogenes and other pseudogenes based on human–macaque orthologs. They identified 34 transcribed pseudogenes, all that have ω significantly smaller than 1 were retrogenes. Two of them, *FUNDC2P* (retro_hsap_2122) and *TCEB2P2* (retro_hsap_940)*,* were also identified in the course of this study as putatively encoding peptides. However, orthology between humans and macaques may not be the best approach for these kinds of studies since a lot of retrocopies are species specific and therefore, many retrocopies present in the human genome will not have any orthologs in macaque or any other primate. In fact, a big part of human protein-coding genes that arose via retroposition are human or hominids specific. Also, as this study has revealed, in some cases peptides matched a retrocopy in a frame that is different from parental gene translation, including reverse direction.

#### 3.3.3. Ribosome Associated Retrocopies

**Figure 4:**
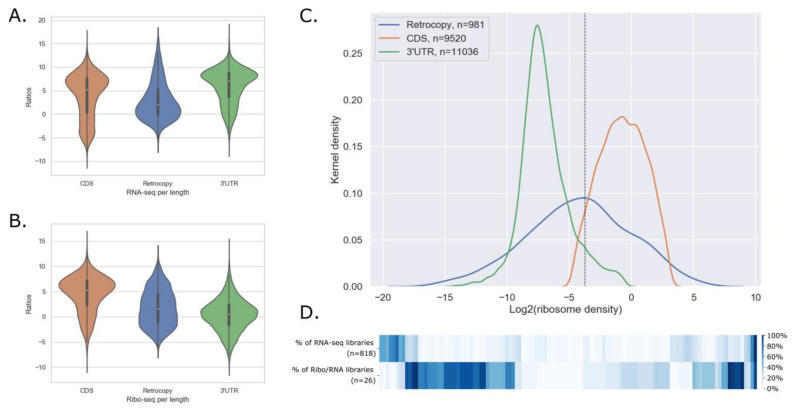
(A) Violin plot for RNA-seq coverage for coding sequences, 3′UTRs of protein-coding genes, and retrogenes in lymphoblastoid cell line as an example. (**B**) Violin plot for Ribo-seq coverage for coding sequences, 3′UTRs of protein-coding genes, and retrogenes in lymphoblastoid cell line. (**C**) Kernel density distribution of ribosome density in lymphoblastoid cell line; dotted black line marks the cut off level calculated based on Z-score of 1.64 in 3′UTR group. (**D**) Heatmap for 117 retrocopies that have a positive signal from expression analysis in 818 ENCODE samples, peptide analysis and ribosome density analysis.

Another way to identify retrocopies that could encode proteins is an analysis of association with ribosomes (Figure 1A). To this end uniquely mapped Ribo-seq and RNA-seq reads from the 26 samples, stored in GWIPs-viz Browser [42], were utilized. Following methodology used by Zeng et al. [44] three groups of sequences were created: coding sequences from all protein-coding genes, 3′UTRs of protein-coding genes, and retrogenes, excluding those annotated as known protein-coding genes. In each library RNA-seq reads coverage and Ribo-seq coverage was calculated. Results were filtered based on RNA-seq coverage and Z-score as described in Materials and Methods. Figure 4 A–C shows, as an example, violin plots demonstrating RNA-seq, Ribo-seq coverage, and Kernel density distribution of ribosome density in one of the analyzed libraries.

Some 1798 retrocopies were identified with a positive signal in at least one library, this makes 38.99% of all retrocopies annotated in the RetrogeneDB database. A total of 757 retrocopies demonstrated association with a ribosome in ten or more libraries. No retrocopy showed association in all libraries; however, in the case of ten retrocopies a strong signal was detected in 24 samples. A percentage of retrocopies with a positive signal for ribosome association is comparable to other studies, although not performed exclusively for retrocopies. Nevertheless, Ji et al. [81] found that out of 426 pseudogenes expressed in libraries studied by them, 155 (36.4%) are translated into peptides longer than 10 aa, and Zeng et al. [44], studying lncRNA, found that a very similar fraction (39.17%) of expressed lncRNA may interact with ribosomes in humans. On the other hand, Guttman et al. [82] argue that the large majority of lincRNAs do not function through encoded proteins.

Considering the abovementioned results and to make this study data more trustworthy, results of both methods, mass spectrometry data analysis and ribosomal profiling, were merged. As many as 359 retrocopies had a signal for peptides coding from both methods, analysis of mass spectrometry data and Ribo-seq. Out of them 117 are also expressed in at least 9 RNA-seq libraries from the 818 ENCODE experiments (Figure 4D, Table S4). It is apparent that retrocopies with a strong ribosome association signal tend to be expressed in a limited number of samples. This may indicate some very specific functions of encoded peptides, restricted to only certain cell types.

One hundred and seventeen identified retrogenes illustrate that a part of annotated as pseudogenes retrocopies may function as a protein or encode short peptides that, as it has been shown, may regulate other genes expression [62]. The fact that some of them have a disrupted ORF, i.e., introduced by mutations stop codons and/or frameshifts, does not exclude a possibility of them playing significant roles. Recent studies on cancer resistance in elephants demonstrated that truncated proteins encoded by multiple retrocopies of the tumor suppressor gene *TP53* may be behind the increased body size, the higher resistance to DNA damage, and a lower incidence of cancer in elephants [12,83]. Still, evidence for peptide coding needs to be interpreted with caution since some retrocopies may be translated into functionally irrelevant peptides [81] and association with the ribosome may be related to transcript degradation [84].

#### 3.3.4. Novel Exons of Protein-Coding Genes

**Figure 5:**
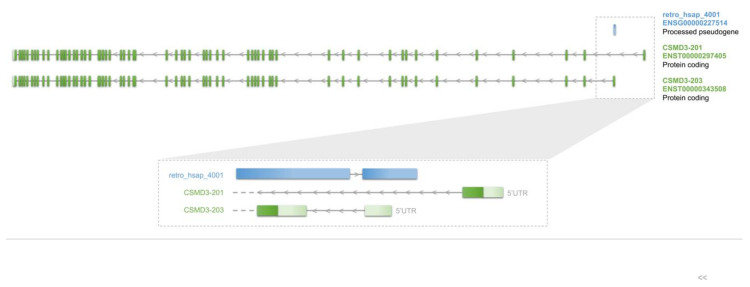
Example of retro_hsap_4001 contribution to novel isoforms of *CSMD3* gene.

Retrocopies may also contribute a novel sequence to already existing genes [21]. Performed analysis revealed 71 transcripts with exapted sequences of 56 retrocopies (Table S5). In 47 cases a retrocopy was incorporated into the gene from the opposite DNA strand. From all 71 identified transcripts only 42 have a complete CDS and were further analyzed. The vast majority of identified retrocopies are integrated in UTRs. Eighteen retrocopies contributed to the coding sequence of the host gene, out of which seven are present exclusively in coding exons. This is not much more than it was found by Baertsch et al. [21], who identified fifteen cases of coding exons derived from a nested retrocopy. This may indicate that retrocopies contribution to novel exons of host genes is rather limited. Nevertheless, integration of these retrocopies contributed toward new splicing forms, often with modified protein and specific expression pattern. For example, retrocopy retro_hsap_4001, located in the gene *CSMD3* locus, contributed to the first two exons of the shorter splice variant with a more upstream transcription start site (TSS) (Figure 5). In the result, the “new” isoform containing retrocopy, does not encode the first 59 amino acids that are present in the longer form. Instead, at the N-terminus of the protein there are nineteen amino acids encoded by the sequence incorporated from the retrocopy. Interestingly, it has been shown that transcripts that use the first exon derived from the retrocopy are expressed in the testis and not in the brain as other splice variants [85]. 

Retrocopies also contributed to the well known gene *BRCA1*. One of the splice variants utilized a part of retro_hsap_2438 as an extra exon. The additional peptide region encoded by the retrocopy-delivered exon might modify the trans-activation domain 1 and the interaction domain of *BRCA1*. We also found three retrocopies that clearly provided conserved domains to protein-coding genes. Retro_hsap_1028 was the source of NAP domain in gene *NAP1L1*, the Keratin 2 domain in gene *TTBK2* was recruited from retro_hsap_1557, and retro_hsap_2219 contributed the HMG box to the *SP100* gene.

### 3.4. Retrocopies as Regulatory Elements

Pseudogenes are considered to be biologically inconsequential because they harbor premature stop codons, deletions/insertions, and frameshift mutations that impede their translation into functional proteins. Nevertheless, nucleotide sequences contained within pseudogenes are often well conserved, implying selective pressure to maintain these genetic elements and that they may play important regulatory roles (Figure 1). The best known example is probably *PTENP1*, a retrocopy of gene *PTEN*, which regulates parental gene expression by competing for miRNA [18]. Another interesting case is a retrocopy of *HMGA1* (high mobility group A1), which is over-expressed in diabetic individuals [86]. The HMGA1 protein regulates the insulin receptor (*INSR)* gene. RNA of the *HMGA1* retrogene competes with *HMGA1* 3′-UTR for a critical RNA stability factor [87]. In addition to the already mentioned function as a competing endogenous RNA (ceRNA), retrocopies might regulate their progenitors acting as trans-NATs [88]. Also, owing to the fact that many retrocopies are nested in or overlapping with other genes, they might function as *cis*-NATs and regulate their host expression via transcription interference or RNA:RNA duplexes, for example.

#### 3.4.1. Competing Endogenous RNAs

As the retrocopies are duplicates of other genes it is conceivable that a number of them may play a similar role to *PTENP1*. The role of retrocopies as competing endogenous RNA, also called miRNA sponges, does not need to be limited to parental genes’ transcripts. Retrocopies may act as ceRNA also for not homologous genes. Performed genome wide analysis revealed that 230 retrocopies may act as miRNA sponges and have a correlated expression with 328 transcripts of 232 genes (Table S6). After filtering and selecting only pairs with a correlation coefficient of |rho| ≥ 0.25, remained 189 transcripts of 181 retrocopies putatively regulating 250 transcripts of 187 genes. Since some retrocopies could act as ceRNA for more than one transcript this resulted in 311 ceRNA-transcript pairs. In the majority the expression correlation between respective transcripts was positive. However, in 53 cases the correlation was negative. Although it is expected that miRNA sponges should have positive expression correlation with regulated genes, we did not remove opposite cases from the summary file. This is due to the fact that in many instances this could result from double functions and/or more complicated networks of ceRNAs and other lncRNA, as demonstrated below. 

Among the identified putative miRNA sponges is the retrocopy of the *NDUFV2* gene, *NDUFV2P1* (retro_hsap_2068), which has been previously shown to have an increased expression in schizophrenia-derived cells [89]. *NDUFV2P1* also demonstrated a significant inverse correlation with *NDUFV2* pre- and a matured protein level. It has been suggested that the *NDUFV2P1* mRNA interferes with the mRNA of *NDUFV2* [89], possibly compiting with the *NDUFV2* at the translation level, which could be yet another way of regulating parental genes by their retrocopies.

Interestingly, in our final set of putative ceRNA the *PTENP1* was missing. This is because the correlation coefficient for the retrogene and the transcript of the parental gene was 0.16, which is below our threshold. This low correlation coefficient may be resulting from the fact that *PTENP1* acts as a decoy for miRNAs, but it is also transcribed in an antisense direction. This antisense lncRNA expressed from the *PTENP1* locus is able to localize to the *PTEN* parent locus and recruit chromatin-remodeling machinery, which leads to the silencing of *PTEN* transcription [18,90].

#### 3.4.2. Trans Natural Antisense Transcripts

Owing to a large number of transcriptomic studies, it is becoming apparent that many pseudogenes, and this includes retropseudogenes, are transcribed into long noncoding RNAs. Some of them have proven biological functions [26,91]. Nevertheless, despite these and other studies, the functions of pseudogene-derived lncRNAs are still an underexplored mechanism of gene regulation that occurs more broadly than previously realized. We have identified 256 lncRNAs that have a sequence derived from retrocopies. 180 retrocopy derived lncRNAs were located on the opposite strand of the DNA. A sequence complementarity to parental genes is also true for 69 transcripts of protein-coding genes which contained exons acquired from retrocopies in antisense orientation. Altogether this gives 249 candidates for trans natural antisense transcripts (trans-NATs) regulating parental genes of respective retrocopies. 

Analysis of the expression in 818 libraries revealed that 78 transcripts, with sequences derived from 52 retrocopies, have expression significantly correlated (*p* < 0.001) with 199 transcripts of 46 retrogenes’ progenitors (Table S7). After filtering out pairs with |rho| < 0.25, there remained 67 transcripts of 45 genes that had expression correlated with 140 transcripts of 40 parental genes. In 104 cases the correlation was positive and in 36 instances negative.

The highest positive correlation, rho = 0.76, was observed for the non-coding transcript of *PRMT1* and protein-coding transcript of gene *NSD1* that contains the *PRMT1* retrocopy retro_hsap_3406. Worth mentioning is probably also the case of the *ATR* gene containing a fragment of retrogene retro_hsap_2713. *ATR* has fifteen isoforms but only one transcript, *ATR.205*, demonstrated a significant expression correlation with the transcript of *EIF2AKI* gene, progenitor of retro_hsap_2713. Transcript *ATR.205* is the only one, which contains an entire retrocopy and therefore has a much longer region complementary to the parental gene than other transcripts.

Additionally, utilizing data from the FANTOM5 project [92], identified were also TSSs (Transcription Start Sites), which are located in the proximity of the 5′ and 3′ ends of annotated retrocopies [33]. Based on this data we pinpointed retrocopies that have a TSS located on the opposite strand and near the 3′ end, which could indicate that these retrocopies are transcribed in an antisense direction and their transcripts are complementary to parental genes transcripts. Therefore, these retrogenes may also act as trans-NATs. We found 47 such retrocopies. One is overlapping with lncRNA located on the opposite strand of DNA and already has been considered as trans-NAT. Out of the remaining 46 retrocopies, twelve revealed significant expression correlation with parent genes transcripts. In the case of seven retrocopies correlation was always negative, two retrocopies had positive expression correlation and in the case of three the correlation was positive with some transcripts and negative with others.

Altogether there were 295 candidates identified for trans-NATs regulating parental genes of respective retrocopies. This is significantly more than in the study by Muro et al., who found 87 transcripts expressed antisense of human pseudogenes [93] and greatly expands the set of 58 transcripts previously identified by us [88]. Out of 295 candidates 78 revealed significant expression correlation with transcripts of retrocopies’ parental genes [94]. Because of a high sequence complementarity these molecules may form RNA:RNA duplexes that play an important role in the modulation of pre-mRNA splicing, RNA editing, mRNA stability control, and abrogation of miRNA-induced repression [95,96]. At the whole transcriptome level, near 60000 transcripts could be regulated in humans by means of lncRNA-RNA interactions [88]. The capacity of retrocopies derived transcripts to act as trans-NAT has been confirmed by some studies. For example, it has been shown that *OCT4* is regulated by a long non-coding RNA antisense to Oct4-pseudogene 5 [96]. Retrogene participates in forming a complex with other RNAs and genomic modifiers to epigenetically modulate DNA transcriptional activity.

#### 3.4.3. *Cis* Antisense Transcripts

As many as 2139 retrocopies overlap with 2071 genes, from which 1301 are located on the opposite strand. This includes overlaps in the exonic as well as in the intronic region of the retrocopies’ counterparts. These retrocopies may regulate the expression of genes they overlap with either at the transcriptional or the post-transcriptional level as it was previously described for antisense transcripts [95,97]. Analysis of expression correlation revealed that 656 retrocopies have correlated expression with 2414 transcripts of 618 genes (Table S8). The strongest correlation was found for retro_hsap_217. This retrocopy of gene *PDIA* has three splicing variants and two of them have expression positively correlated with the longest transcript of gene *FMO5,* in which the retrocopy is embedded. The correlation coefficient for *PDIA3P1-201* is 0.89 and for *PDIA3P1-202* is 0.80. A very strong correlation, rho = 0.82, was also found for another retrocopy also embedded in the same *FMO5* gene, retrocopy of the *RPL7A* gene (retro_hsap_218). Interestingly, the host gene *FMO5* as well as *PDIA3P1* are both associated with cancer [98,99].

An interesting example coming out of this study is retro_hsap_4762, a retrocopy of gene *RAB28*, which overlaps with transcripts of two genes and has a positive expression correlation with both of them. One of these genes is *RBMX* associated with Shashi X-linked mental retardation syndrome [100] and also identified as component of the DNA-damage response [101]. Another retrocopy, retro_hsap_1259, a duplicate of gene *RCN1*, possibly regulates the expression of the *TPT1-AS1* transcript, an lncRNA downregulating the microRNA-770-5p to inhibit glioma cell autophagy and promote proliferation through *STMN1* upregulation [102].

Antisense transcription is a quite common way of expression regulation [103]. We identified as many as 657 retrocopies that are in antisense orientation to their host genes and have significant expression correlation with some of these genes’ transcripts. Therefore, it is plausible that identified retrocopies do regulate the level of their hosts’ expression. 

#### 3.4.4. Splicing Regulation by Transcriptional Interference

**Figure 6:**
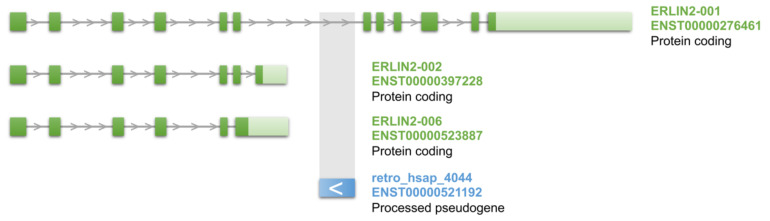
Three splicing variants of *ERLIN2* gene (marked in green) and nested in the intron retrocopy (marked in blue). The retrocopy is located on the opposite DNA strand and according to Kaer et al. [104] expression of the retrocopy may facilitate early transcription termination and emergence of shorter transcripts.

Kaer et al. [103] investigated several coding and noncoding genes nested in the intronic region of another gene and found that nested genes cause premature termination of host gene transcripts by forced exonisation of the intronic region and providing alternative polyadenylation signals. Premature termination of transcription may be induced by mechanisms of transcriptional interference like “sitting duck” or polymerase collision [95]. Our analysis of retrocopies nested in other genes revealed 50 retrocopies localized no more than 1000 bp downstream of one isoform and in the intron of another splicing form of the same gene. An example of such genes arrangements is demonstrated in Figure 6. 

Twenty eight of the identified retrocopies were in the orientation opposite to the host gene and 22 in the same orientation. Out of these, seventeen demonstrated significant expression correlation with a shorter transcript (or transcripts) of the host gene but not with the longer ones. In all of these cases the expression correlation was positive and therefore the higher the level of retrocopy transcription was, the more isoforms with the shorter sequence were observed. In nine instances the retrocopies are annotated on opposite and in eight on the same strand as the host gene. Polymerase collision is the most common mechanism of transcription interference and it occurs in the case of divergent transcription. Nevertheless, in the case of convergent transcription some other mechanisms, e.g., sitting duck interference, occlusion or roadblock, may be involved [105]. 

The highest correlation between short isoform and nested retrocopy, over 0.7, was revealed for the retrocopy of gene *EIF1* (retro_hsap_1659) and two short splicing forms of *PRSS21,* a metastasis-associated ovarian cancer gene [106]. An interesting example is *ERLIN2* and the embedded in this gene retrocopy of *CXorf56* (retro_hsap_4044) (Figure 6). The retrocopy has a positive expression correlation with two short splice variants of *ERLIN2*. The correlation is quite strong, 0.63 for one short transcript and 0.58 for another. *ERLIN2* was associated with metastasis in breast cancer [107]. Also, mutations in *ERLIN2* cause the neurologic disorder spastic paraplegia type 18 [108], lateral sclerosis [109], and mental retardation [110]. Interestingly, *CXorf56*, a parental gene of retro_hsap_4044, was also associated with intellectual disability [111]. Both genes encode endoplasmic reticulum-associated proteins [112,113]. In addition, as our analyses revealed, retro_hsap_4044 may regulate its own parental gene acting as a miRNA sponge, although the expression correlation coefficient is slightly below our cut off, rho = 0.24. Obviously functional links between these three genes need to be confirmed experimentally. 

#### 3.4.5. Regulatory Networks

The abovementioned example of the *PTENP1* retrocopy is an excellent representation of lncRNAs dual functions having an opposite effect on parental gene expression. In the course of this study more instances of retrocopies that possibly play more than one function were identified. Also, pinpointed were parental genes, which are putatively regulated by more than one retrocopy and the expression correlation was with some retrocopies positive and with other negative. Moreover, some retrocopies may regulate expression of more than one gene. All of this data provides evidence that a number of retrocopies may be involved in complex regulatory networks. 

**Figure 7:**
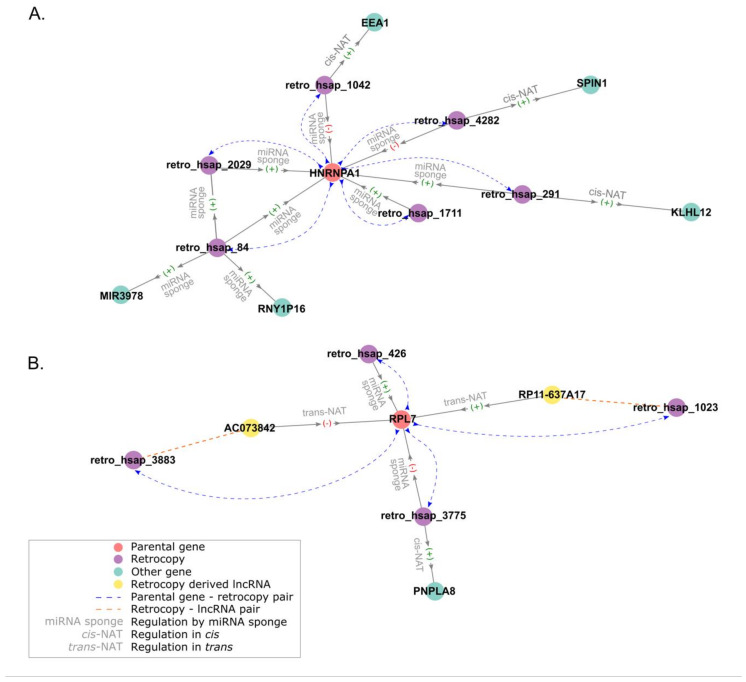
Hypothetical regulatory networks involving retrocopies of (**A**) gene *HNRPA1* and (**B**) gene *RPL7*.

Figure 7 shows an example of two hypothetical networks. In the first example six retrocopies are regulating their progenitor *HNRNPA1*. All of the retrocopies have conserved target sites and may sequestrate miRNA that are targeting the parental gene. However, in three cases the correlation expression is positive and in three it is negative. Three of these retrocopies are nested in other genes and may act as *cis*-NATs. All of them have positive expression correlation with the host gene but only one also has a positive expression correlation with the parental gene. The remaining two nested retrocopies have a negative correlation with *HNRNPA1* transcripts. Interestingly, another retrocopy (retro_hsap_84) is a known protein-coding gene *HNRNPA1L2* and might also compete with *HNRNPA1* for miRNA. In addition, it can act as ceRNA for two not homologous genes. One of them is a retrocopy of yet another gene, *RNY.*

Figure 7B represents gene *RPL7*, which can be regulated by four transcripts. In two cases these are lncRNAs transcribed from the opposite to the retrocopy DNA strand, acting as trans-NATs, and in two cases retrocopies may play a role of miRNA sponges, although one has a positive correlation with the parental gene and another negative. The one that has a negative correlation with *RPL7* is embedded in gene *PNPLA8* and, since its expression is correlated with the host’s transcript, it is possible that it acts as a *cis*-NAT. 

#### 3.4.6. Functional Evolution—A Case Study of retro_hsap_1589

Rertro_hsap_1589 is a retrocopy of a high mobility group box 1 gene (*HMGB1*). The retrocopy is expressed in 781 libraries with an average expression over 24 reads per transcript. It demonstrates 98% similarity to the parental gene and was identified, in the course of this study, as lncRNA putatively sequestrating miRNA targeting its progenitor. *HMGB1* is ubiquitously expressed and is encoding a nuclear DNA-binding protein that regulates transcription of many genes and is involved in the organization of DNA. This protein plays a role in several cellular processes, including inflammation, cell differentiation, and tumor cell migration and is associated with numerous diseases [114,115,116,117]. Therefore, the retrocopy acting as ceRNA and regulating *HMGB1* gene expression may play a vital role in many biological processes. This may explain the ubiquitous expression of retro_hsap_1589.

**Figure 8:**
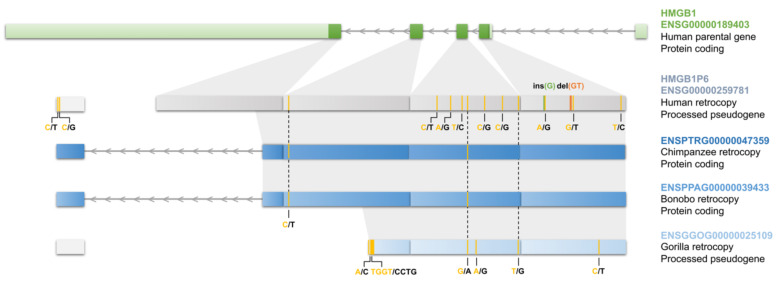
Comparison of retrocopy retro_hsap_1589 in humans and its orthologs in chimpanzees, bonobos and gorillas.

The retroposition of this gene occurred in the ancestors of *Homininae* (African apes) and in the ENSEMBL database it is annotated as a processed pseudogene. However, its orthologs in chimpanzees and bonobos are marked as protein-coding genes. In gorillas, similarly to humans, this is a pseudogene. Analysis of retrocopies sequences revealed that several mutations, including one deletion and one insertion, occurred in the human retrogene after the divergence of the human lineage (Figure 8). In bonobos and chimpanzees there were no mutations, and, in addition, one exon was gained at the 3′ end. Therefore, in these two species, the protein encoded by retrogenes differs at the C-terminus from the protein encoded by parental genes. This is a perfect example of species-specific contribution to the transcriptome. After a single event of retrotransposition in the ancestor of African apes’, the retrocopy evolved differently in various species. While in chimpanzees and gorillas this retrocopy was relatively quickly under a negative selection and codes for a protein which possibly shares a function with the parental gene, in humans a number of mutations accumulated and the retrogene acts as a lncRNA. Considering its high and broad expression in human, its functions may be indispensable.

### 3.5. Retrocopies as Recombination Hot Spots

**Figure 9:**
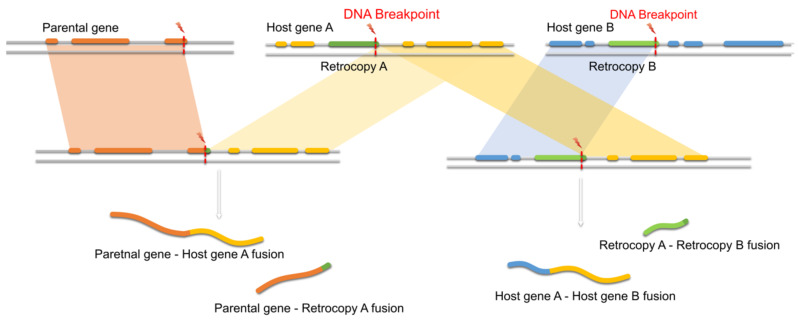
Possible outcomes of recombination events with the involvement of retrocopies.

Our analysis revealed that gene *ATR* contains a sequence of retro_hsap_2713 and may act as a *trans*-NAT for the retrocopy’s parental gene. Interestingly, fusion transcripts containing exons of genes *ATR* and *EIF2AK1,* a parental gene of retro_hsap_2713, have been found in cancer cells. It was proposed that this fusion results from a recombination event between chromosomes 7 and 3 [118]. It is quite plausible that this recombination was stimulated by the high sequence similarity between the parental gene and its retrocopy embedded in gene *ATR*. Such non-allelic homologous recombination between a retrocopy and its parent, or two retrocopies of the same gene, could be considered as an additional contribution of retroposed genes to genome and transcriptome evolution, the same way as it is in the case of transposable elements [119,120,121]. Fusion transcripts resulting from such chromosomal rearrangements may be formed by parental genes, genes hosting respective retrocopies or the retrocopies themselves (Figure 9).

To investigate this, data from the FusionGDB database [54] was analyzed. Nineteen fusion transcripts resulting from chromosomal rearrangements and indicating involvement of retrocopies were identified (Table S9). In thirteen instances the fusion transcript contained exons of the parental gene and exons of its retrocopy host. Two transcripts represented a fusion between a retrocopy and its respective progenitors and in three cases the fusion transcript was made up of exons of two genes hosting retrocopies of the same gene. No example of a fusion transcript that was build up from two retrocopies has been pinpointed. However, an interesting case was found where a fusion transcript was formed by the microRNA gene *MIR7111* embedded in the *RPL10A* gene and the retrocopy of *RPL10A* embedded in gene *PLD5*.

An analysis of breakpoints revealed that in fifteen cases the breakpoint was in the body of the retrocopy. In four instances a chromosome broke downstream however, only 20 to 210 bp away from the annotated span of the retrocopy. Only in one case, involving a retrocopy of gene *TUBG1*, the chromosome broke over 6000 bp from the retrocopy. Still, we cannot exclude the retrogene involvement in the formation of the recombination spot since the transcript is build up from the parental gene *TUBG1* and a host of its retrocopy. One of the genes forming fusion transcript, the famous speech gene *FOXP2* associated with language and speech disorders, hosts a retrocopy of gene *RPL36*. It has been found that this gene forms fusion transcripts not only with *RPL36* but also *COG5*, *RCF3*, and *SFTBP* [122].

The abovementioned examples demonstrate that although most retrocopies likely represent dead-on-arrival gene copies that have lost both protein-coding capability and transcriptional activity, they may still contribute to the evolution of genomes. Full-length coding sequences of retroposed genes merged with other transcribed sequences have been previously reported [20,21,123]. However, these were mostly cases of retrocopy fusion with a nearby gene or a non-genic sequence. Here, to the best of our knowledge, we report for the first time fusion transcripts resulting from chromosomal rearrangements involving retrocopies as recombination hot spots.

## 4. Conclusions

Evolutionary trajectories of retrocopies include a range of possible outcomes, making the distinction between not functional retrocopies and retrogenes quite difficult. Analyses aiming at identification of functional retrogenes were for a long time based on ORF conservation. The occurrence of an intact open reading frame with a length similar to the coding sequence of the parental gene, dN/dS ratio significantly lower than 1, and expression in at least one tissue was usually required to consider a retrocopy as functional. This was based on the assumption that functional retrocopies code proteins and their function does not differ much from the function of their progenitors. Functional genomic and transcriptomic data generated over the last decades revealed that hundreds of retrocopies are transcriptionally active [6,33,124,125,126] and ORF conservation is not necessary for a retrocopy to be functional. Nevertheless, despite these and other publications, the functions of retrocopies are still an underexplored mechanism of gene regulation, which occurs more broadly than previously realized. The results of our studies and examples provided in this manuscript illustrate that neither ORF disruption nor presumed loss of promoter activity upon retroposition proves that a gene is nonfunctional. They also illustrate that retroposition of protein-coding genes had a profound impact on the human genome, proteome, and transcriptome.

## Figures and Tables

**Table 1:** Retrocopies with a ubiquitous and tissue specific expression.

Type of Expression	Number of Retrocopies	Identifiers from RetrogeneDB
Ubiquitous	14	retro_hsap_2, retro_hsap_4, retro_hsap_36, retro_hsap_57, retro_hsap_64, retro_hsap_75, retro_hsap_100, retro_hsap_105, retro_hsap_108, retro_hsap_217, retro_hsap_774, retro_hsap_901, retro_hsap_1605, retro_hsap_3990
All cancer cell lines but not normal tissue	3	retro_hsap_1725, retro_hsap_1817, retro_hsap_2646
**Restricted to a specific tissue type**		
Fetal brain	5	retro_hsap_912, retro_hsap_913, retro_hsap_1813, retro_hsap_1883, retro_hsap_2045
Heart and aorta	2	retro_hsap_316, retro_hsap_3488
Liver	2	retro_hsap_623, retro_hsap_4127
Lung	2	retro_hsap_3266, retro_hsap_4877
Omental fat pad	1	retro_hsap_2759
Peyer’s patch	1	retro_hsap_25
Prostate gland	5	retro_hsap_101, retro_hsap_743, retro_hsap_770, retro_hsap_2122, retro_hsap_4833
Skin	8	retro_hsap_178, retro_hsap_734, retro_hsap_1483, retro_hsap_1713, retro_hsap_2147, retro_hsap_2266, retro_hsap_3080, retro_hsap_3112
Spleen	14	retro_hsap_241, retro_hsap_396, retro_hsap_671, retro_hsap_877, retro_hsap_1801, retro_hsap_2073, retro_hsap_2092, retro_hsap_2576, retro_hsap_2666, retro_hsap_2799, retro_hsap_3524, retro_hsap_3613, retro_hsap_3678, retro_hsap_3917
Tibial nerve	1	retro_hsap_4800
Transverse colon	2	retro_hsap_2044, retro_hsap_4063
Uterus	1	retro_hsap_4139

## Supplementary Materials

The following are available online at https://www.mdpi.com/2073-4425/11/5/542/s1, Figure S1: PANTHER GO-Slim Analysis of protein-coding retrogenes and parental genes. Table S1: Set of analyzed retrocopies. Table S2: Retrocopies expression levels (TPM). Table S3: Protein-coding retrogenes. Table S4: Results of peptides and ribosome association analyses. Table S5: Retrocopies contributing to protein-coding transcripts of other genes. Table S6: Retrocopies as potential miRNA sponges. Table S7: Transcripts overlapping with retrocopies (potential *trans*-NATs) and parental genes’ transcripts. Table S8: Retrocopies as potential *cis-*NATs. Table S9: A. Host gene and parental gene fusions; B. Host gene A and host gene B fusions. Nested retrocopies originated from the same parental gene.
