# Supplementary figures and images for: Complex Analysis of Retroposed Genes’ Contribution to Human Genome, Proteome and Transcriptome
Source: Genes (Basel). 2020 May 12;11(5):542. doi: 10.3390/genes11050542 (PMC7290577; doi:10.3390/genes11050542)

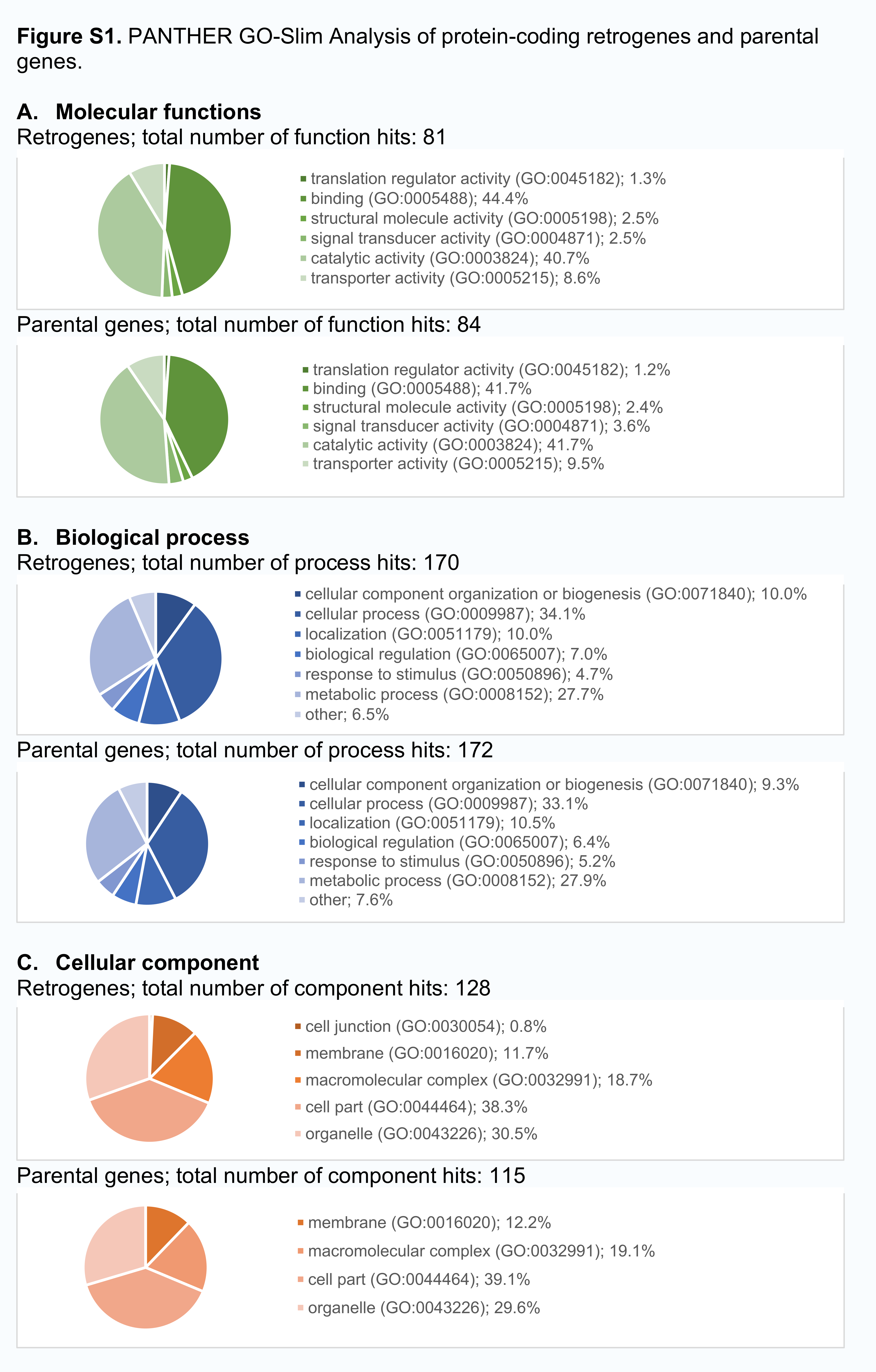

Supplement: Supplementary file 1 [file genes-11-00542-s001.zip › Figure_S1.png]
